# Comparison of the effects of preemptive acetaminophen, ibuprofen, and meloxicam on pain after separator placement: a randomized clinical trial

**DOI:** 10.1186/s40510-015-0104-y

**Published:** 2015-10-14

**Authors:** Hooman Zarif Najafi, Morteza Oshagh, Parisa Salehi, Neda Babanouri, Sepideh Torkan

**Affiliations:** Orthodontic Research Center, Orthodontics Department, School of Dentistry, Shiraz University of Medical Sciences, Shiraz, Iran; Private Practice, Tehran, Iran; Orthodontics Department, University of Washington, Seattle, WA USA

**Keywords:** Orthodontic pain, Non-selective NSAIDs, Meloxicam

## Abstract

**Background:**

This study aims to evaluate and compare the effect of pre-procedural administration of acetaminophen, ibuprofen, and meloxicam in reducing pain after separator placement.

**Methods:**

Three hundred twenty-one patients who needed orthodontic treatment and aged above 15 were randomly assigned to one of the three study groups: group A: 650 mg acetaminophen, group B: 400 mg ibuprofen, and group C: 7.5 mg meloxicam. All subjects received a single dose of medication 1 h prior to separator placement. Using visual analog scale, patients recorded their pain perception during rest, fitting posterior teeth together, and chewing at time intervals of immediately, 2, 6, 24, and 48 h after separator placement.

**Results:**

There was no significant difference between acetaminophen, ibuprofen, and meloxicam in post-separator placement pain control when administered 1 h before the procedure. In all the groups, at rest, pain level elevated after separator placement and reached its peak at 24 h and then subsided until 48 h. But during chewing and fitting of the posterior teeth, some of the groups reached a peak in pain at 48 h. No significant difference was found in pain experience between males and females.

**Conclusions:**

Meloxicam can be used as an effective analgesic in orthodontic pain control considering it has less gastric side effects compared to the conventional nonsteroidal anti-inflammatory drugs.

**Trial registration:**

Iranian Registry of Clinical Trials, IRCT2015041821828N1

## Background

Pain is an unpleasant sensation caused by some tissue changes. These tissue changes in orthodontic treatment are caused by the compression of periodontal ligament and alteration of blood flow to the tooth, resulting in releasing chemical mediators like prostaglandins [1, 2]. About 95 % of the patients undergoing orthodontic therapy report varying degrees of pain and discomfort during some stages of treatment such as separator or arch wire placement [3, 4]. It has also been reported that pain is the foremost reason for patient aversion and discontinuing treatment [5, 6]. It has been claimed that degree of pain experienced by patient varies based on gender, age, patient anxiety level, and emotional stress [1, 4, 7, 8].

Despite the concerns stated by the orthodontists and patients, no standard of care has still been defined to control pain caused by orthodontic appliances [9, 10]. Several methods have been proposed such as administration of analgesics, introducing vibratory stimulation, chewing on a bite wafer, and most recently, the use of low-level laser therapy [11–22].

Analgesics are the most common treatment modality used to control the pain associated with orthodontic treatment [15]. Several studies evaluated the effect of pre- and postoperative use of various medications including aspirin, acetaminophen, ibuprofen, piroxicam, etc. on the orthodontic-induced pain [11–18, 20, 23]. Acetaminophen is an over-the-counter medication with antipyretic and analgesic effects via central inhibition of the third isoform of cyclooxygenase enzyme (COX_3_), which is mostly found in the cerebral cortex and heart [13, 24]. The conventional nonsteroidal anti-inflammatory drugs (NSAIDs) like ibuprofen, piroxicam, aspirin and naproxen sodium block the production of prostaglandins through inhibiting the other isoforms of cyclooxygenase (COX) enzyme [24].These medications are called non-selective COX inhibitors, since they block both COX_1_ and COX_2_ isoforms [24]. Inhibition of COX_1_ is responsible for the adverse effects of NSAIDs such as gastric ulceration and bleeding disorders [25]. Many studies have been conducted to evaluate the effects of this family of NSAIDs on the pain relief during orthodontics treatment [11–18]. Bird et al. reported that there was no difference between the single preemptive use of acetaminophen and ibuprofen in pain control after separator placement whereas Bradley et al. showed that ibuprofen was more effective than acetaminophen [13, 14].

Another class of NSAIDs that has been recently introduced is selective COX_2_ inhibitors (coxibs) like celecoxib, valdecoxib, and lumiracoxib [26]. They retain the benefits of anti-inflammatory action with minimum side effects like gastric irritation and platelet functional alteration, and also, they have longer dose interval [25–28]. Young et al. showed that pre- and postoperative use of valdecoxib compared to placebo can effectively decrease pain after archwire placement, while Bruno et al. found no significant difference in post-separator pain between placebo and lumiracoxib [29, 30].

Meloxicam is one of most popular relatively selective COX_2_ inhibitors used in the treatment of acute and chronic inflammatory painful disorders like rheumatoid arthritis, dental pain, and postoperative pain [31–33]. It is now clear that meloxicam has a lower gastric effect compared to other NSAIDs [31]. Efficacy of this drug in controlling post-endodontic pain and pain after third molar removal and oral surgery has been investigated previously [27, 32, 33].

Recently, there have been some concerns regarding the increased risk of cardiovascular and renal events associated with administration of selective COX_2_ inhibitors such as valdecoxib, rofecoxib, and lumiracoxib [9, 30]. COX_2_ inhibitors decrease the production of vascular prostaglandin I_2_ (PGI_2_) which is a vasodilator and anti-aggregator mediator [34]. In addition, COX_2_ inhibitors do not inhibit thromboxane A_2_ (TxA_2_) production, one of COX_1_ products from arachidonic acid in platelets which causes irreversible platelet aggregation, vasoconstriction, and smooth muscle proliferation [34]. However, available data and systematic reviews suggest that meloxicam has more desirable cardiovascular and renal safety profile than other COX_2_ inhibitors like celecoxib and rofecoxib [35].

Considering that only few studies have evaluated the effect of selective COX inhibitors on orthodontic pain control and no studies have evaluated the effect of meloxicam as a relatively selective COX_2_ inhibitor in orthodontic pain control and given the adverse effects of conventional NSAIDs, the authors of this study designed this double-blinded parallel arm randomized clinical trial study to compare the effect of preemptive administration of meloxicam with acetaminophen and ibuprofen on the experienced pain following orthodontic separator placement.

## Methods

The sample size was determined to be 70 in each group based on the mean pain scores recorded in the similar study (acetaminophen (31.6 ± 18.8) and ibuprofen (22.8 ± 17.7)) at *α* = 0.05 and power = 80 % [13]. One hundred seven patients were recruited in this study to account for the potential patient dropouts during the course of the study. Three hundred twenty-one patients who needed fixed orthodontic therapy and were referred to the Orthodontic Clinic of Dental School at Shiraz University of Medical Sciences, Iran, were selected for this prospective double-blind randomized clinical trial investigation. This study was approved by the ethical committee of Shiraz University of Medical Sciences. All participants had the following criteria:

1. Need separator placement to begin orthodontic treatment in the maxillary arch
2. Aged 15 years or older
3. Were informed and signed the written informed consent
4. Not currently using antibiotics, analgesics, anti-inflammatory, anti-coagulative, diuretics, oral anti diabetics, lithium, cyclosporine, and methotrexate
5. No need for antibiotic prophylaxis
6. No chronic systemic disease or clotting disorders
7. Not reporting contraindication for NSAIDs
8. Not pregnant or nursing

The block randomization method was used with block length 9, and number of repetition for each group *m* = 3, to allocate subjects in each group. This method was used separately for each sex group to provide groups with equal numbers of male and female. The patients were divided to three equal groups which were consisted of 78 women and 29 men: group A (650 mg acetaminophen), group B (400 mg ibuprofen) and group C (7.5 mg meloxicam (7.5 mg; BohringerIngelheim Pharms, Germany)). In each group, all tablets were covered by identical gelatin cover, so the investigators, the patients, and the statistician were all blind to the treatment groups. All patients were given only one tablet, 1 h before separator placement (Alastiks S-2separator modules; lot number A2508, 3M UnitekMonorvia, Calif) .The time and the quadrant in which the separators were placed were recorded.

A visual analog scale (VAS) was used to determine the level of pain and discomfort at the following intervals: immediately after separator placement (T_0_), 2 h post-treatment (T_1_), 6 h post-treatment (T_2_), 24 h post-treatment (T_3_), and 48 h after separator placement (T_4_). Each patient received a booklet consisted of five series of VAS. The VAS format was a 10 cm line from 0 indicating no pain to 10 indicating the worst pain a patient has ever experienced. Patients were instructed to mark the degree of the pain with a short vertical line on the VAS during three oral situations including rest, fitting posterior teeth, and chewing, and return the questionnaire in the next visit (a week later). While fitting the posterior teeth, the patients were instructed to bring the teeth together with a light force and not to eat anything in the process. For the chewing function, the subjects were instructed to chew on a slice of granny smith apple and mark the level of the subsequent pain on the VAS. The patients were asked to not use other analgesics during the period of the study, and in case they did so, they would be excluded from the investigation. Before this study, a pilot study was carried out with 20 patients who needed to have separators placed prior to orthodontic treatment. The patients did not take any medications before the treatment. They were asked to record their pain score according to the instructions, and their functions were evaluated via the questionnaire. This pilot study was just performed to ensure the ease of comprehending the instructions by the participants, and none of the subjects were included in the main study.

### Statistical analysis

Statistical analysis was done using the Statistical Package for Social Sciences (Version 15.0, SPSS Inc., Chicago, Illinois, USA). Descriptive analysis was performed for pain scores in three treatment groups for all different time intervals, at rest, fitting the posterior teeth, and chewing. The normal distribution of the data was tested with Kolmogorov Smirnov normality test, before application of parametric tests. Differences in the mean pain score between experimental groups were evaluated by analysis of variance (ANOVA) and Tukey test. Repeated measure ANOVA and paired *t* test was conducted to determine the difference in the pain scores at each time interval. The level of significance for all tests in our study was set at *p* < 0.05.

## Results

**Fig. 1:**
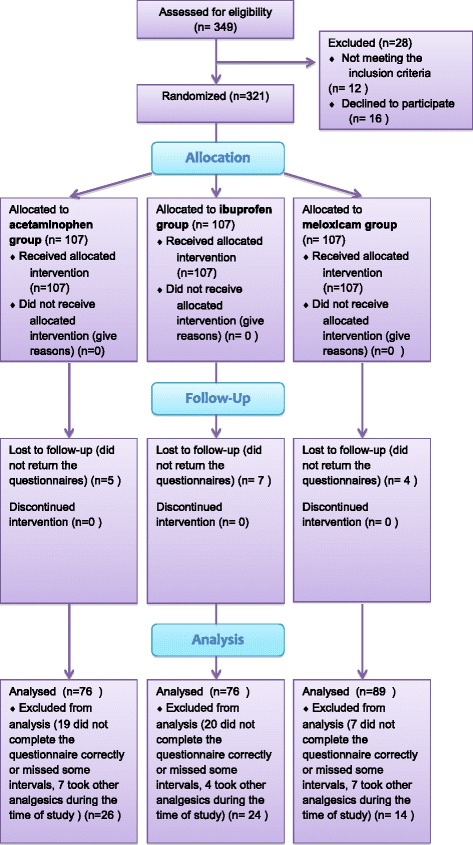
CONSORT flow diagram

Eighty patients (25 %) were dropped out of the study, of whom 18 patients used other analgesics during the time of study, 46 patients did not complete the questionnaires correctly, and 16 did not return the questionnaires. The final sample consisted of 60 men (25 %) and 181 women (75 %), of whom 57 women and 19 men were in the acetaminophen group, 55 women and 21 men in the ibuprofen group, and 68 women and 21 men in the meloxicam group (Fig. 1). No statistical difference was found between the three study groups in terms of age; the mean age of the acetaminophen group was 21.7 ± 3.5 and a mean age of 22.1 ± 3.2 and 21.2 ± 3.8 were recorded for the ibuprofen and the meloxicam groups, respectively. Table 1 outlines the descriptive information and ANOVA results for the three groups.

**Table 1:** Descriptive information and ANOVA results of three treatment groups

Function	Group	T0	T1	T2	T3	T4
Rest	Acetaminophen	0.58 ± 0.97	0.78 ± 1.26	1.08 ± 1.50	1.48 ± 1.91	1.20 ± 2.01
	Ibuprofen	0.54 ± 1.05	0.69 ± 1.30	0.95 ± 1.66	1.78 ± 2.43	1.71 ± 2.24
	Meloxicam	0.66 ± 0.93	0.80 ± 1.16	1.13 ± 1.58	1.42 ± 1.98	1.33 ± 1.84
	*P* value^a^	0.739	0.844	0.758	0.532	0.276
Fitting posterior teeth	Acetaminophen	0.84 ± 1.24	1.13 ± 1.26	1.65 ± 1.69	2.65 ± 2.68	2.10 ± 2.68
	Ibuprofen	0.69 ± 1.10	1.06 ± 1.84	1.48 ± 2.04	2.89 ± 3.00	3.02 ± 2.86
	Meloxicam	0.99 ± 1.28	1.27 ± 1.52	1.73 ± 2.06	2.44 ± 2.48	2.35 ± 2.54
	*P* value^a^	0.281	0.675	0.694	0.566	0.095
Chewing	Acetaminophen	1.01 ± 1.46	1.36 ± 1.56	1.96 ± 1.84	3.08 ± 2.97	2.79 ± 3.17
	Ibuprofen	0.98 ± 1.52	1.37 ± 2.15	1.89 ± 2.42	3.63 ± 3.24	3.75 ± 3.29
	Meloxicam	1.22 ± 1.65	1.50 ± 1.86	2.01 ± 2.14	3.05 ± 2.81	3.16 ± 3.10
	*P* value^a^	0.560	0.871	0.939	0.397	0.177

T0 (immediately after separator placement), T1 (2 h), T2 (6 h), T3 (24 h), T4 (48 h)

^**a**^ANOVA results

Although, there was no statistically significant difference in the pain perception scores between the three treatment groups (Table 1), patients consistently experienced more pain on T_0_ and T_1_ in the meloxicam group and on T_3_ and T_4_ in the ibuprofen group. Additionally, when considering the peak pain scores, the highest values were in the ibuprofen group during the rest, and two masticatory functions and the lowest ones were seen in the meloxicam group during rest and fitting posterior teeth. In the chewing function, the difference between the mean pain score of the three groups tended to be significant 48 h after separator placement (*P* = 0.095). Tukey test showed the difference was between the ibuprofen and the acetaminophen groups (*P* = 0.091). Although, in all study groups and at all time intervals, subjects experienced more pain when chewing compared to at rest and while fitting posterior teeth, but the difference was not significant. Our results also showed that gender had no significant effect on pain perception scores in any treatment groups and for any time intervals.

**Fig. 2:**
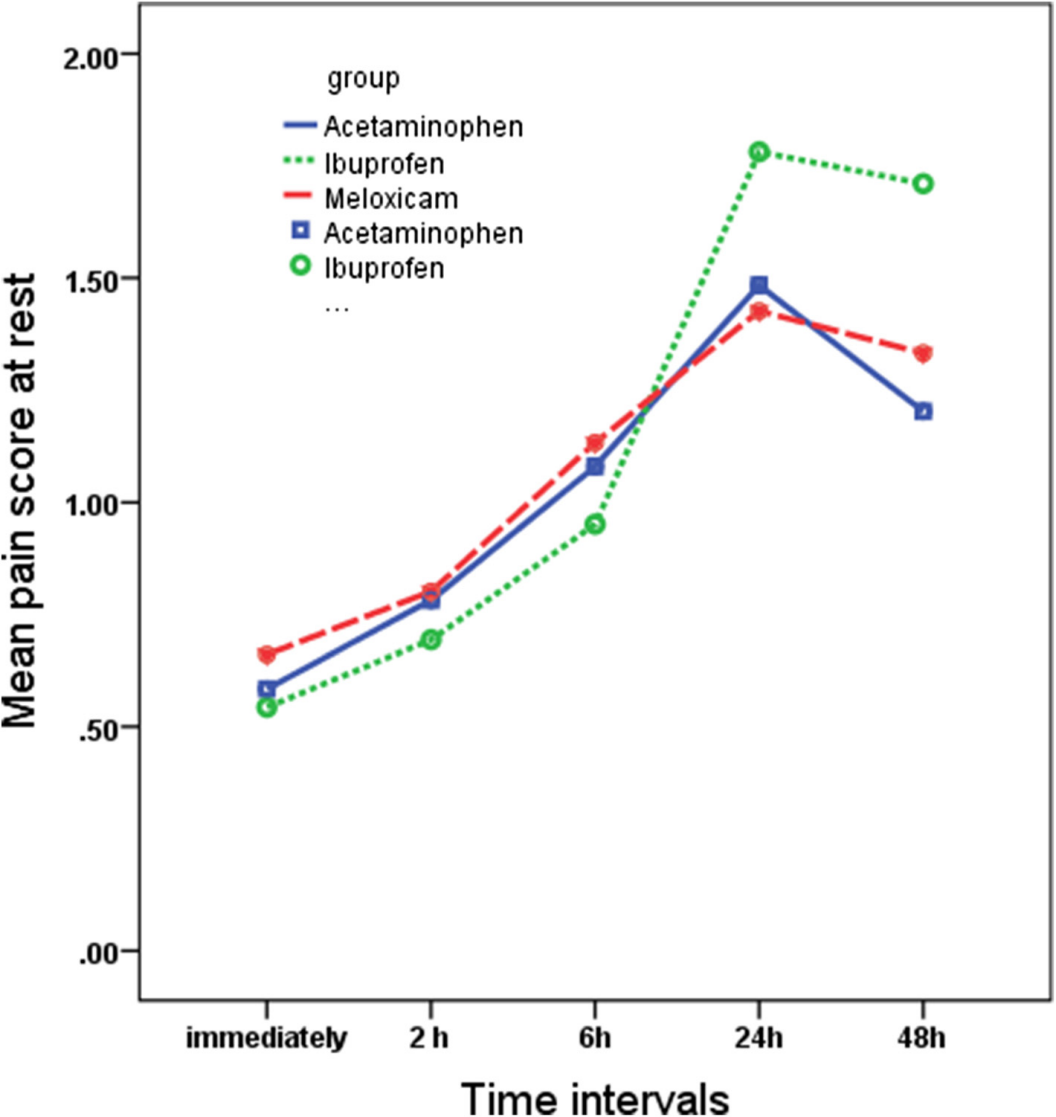
Comparison of the mean pain scores on VAS among the three study groups over the time in the rest position

**Fig. 3:**
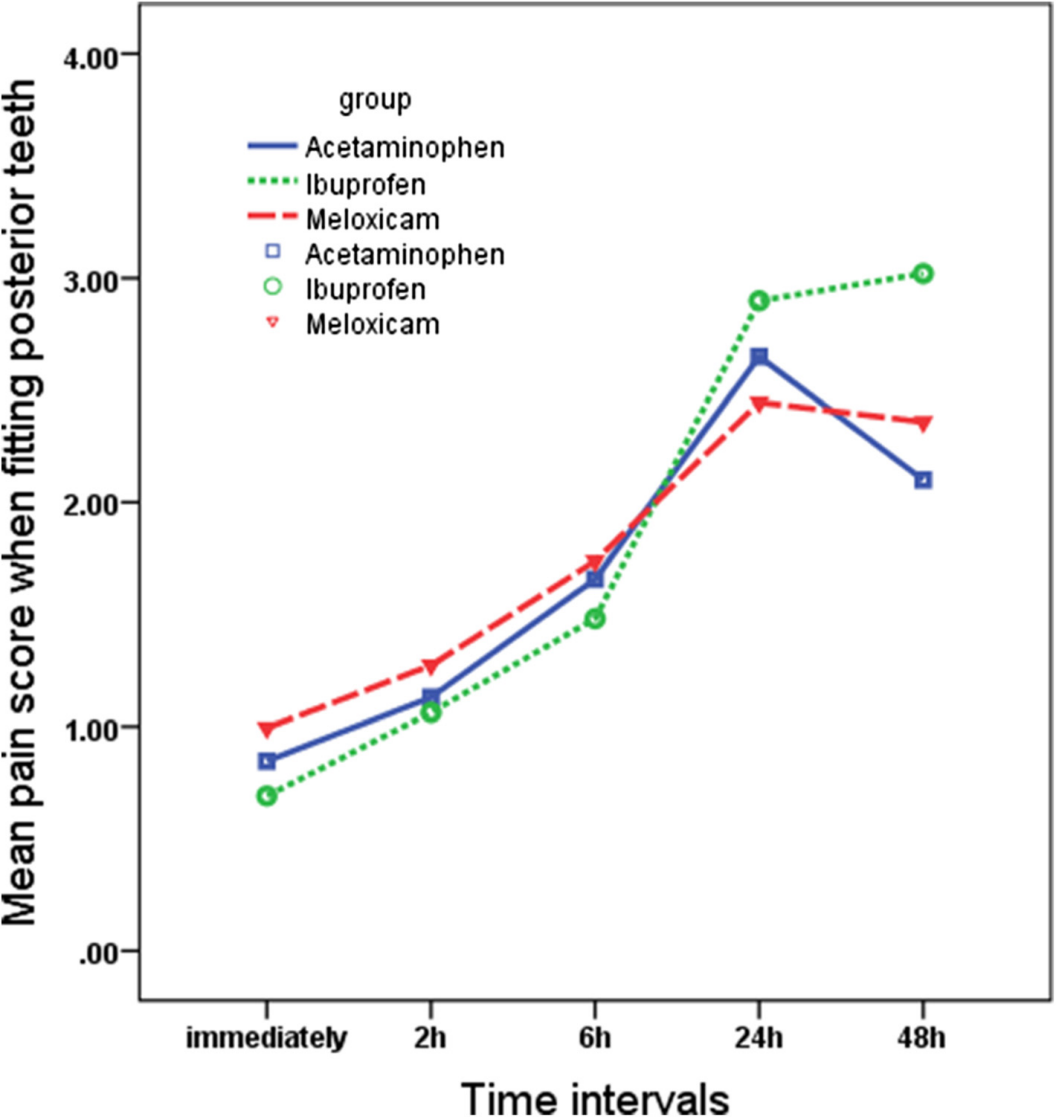
Comparison of the mean pain scores on VAS among the three study groups over the time when fitting posterior teeth

**Fig. 4:**
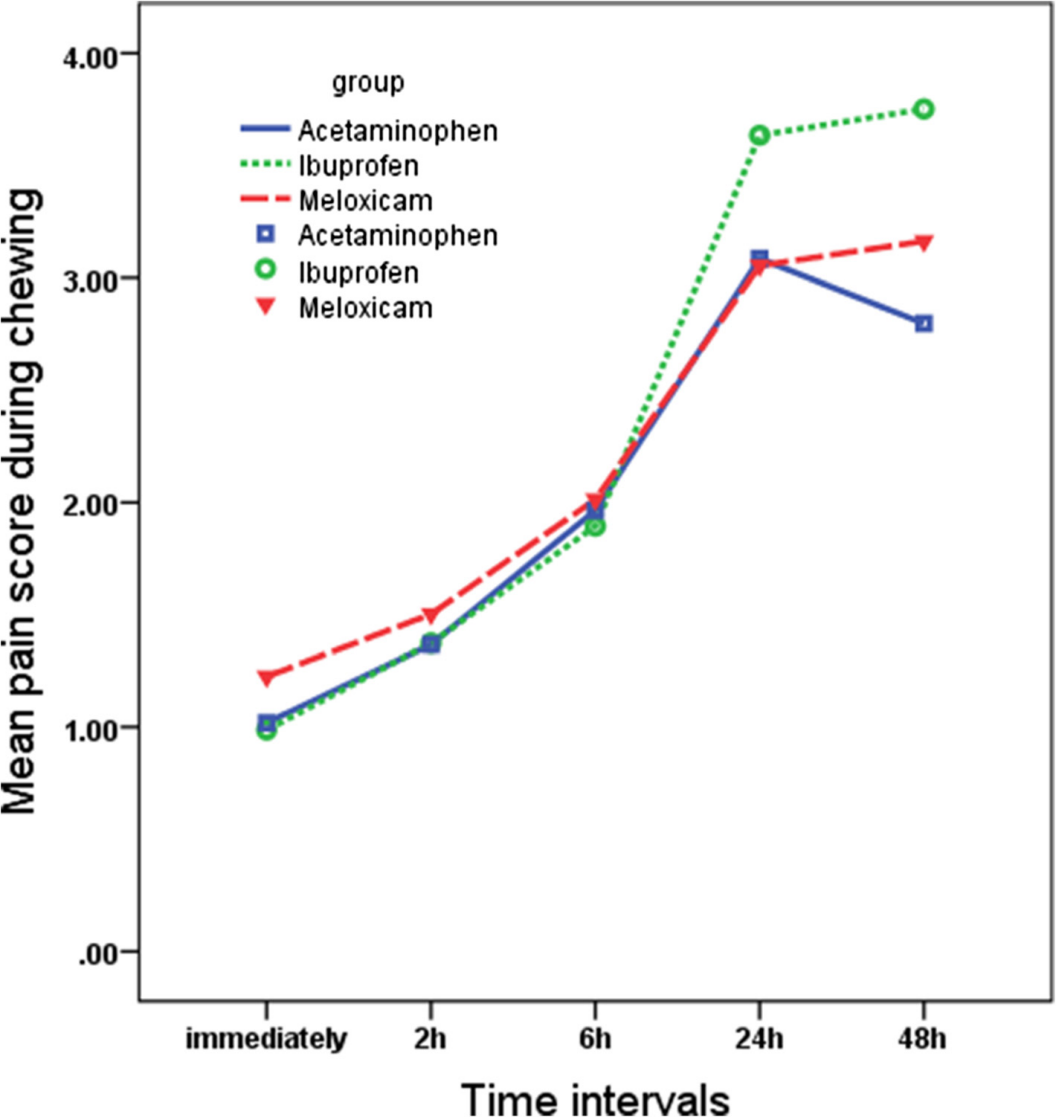
Comparison of the mean pain scores on VAS among the three study groups over the time in chewing function

Significant differences were found in pain perception scores at different times (*P* = 0.001), although the trend was almost similar in all groups and for all masticatory functions (Figs. 2, 3, and 4). The changes in pain perception with the time are presented individually for each medication.

1. *Acetaminophen*. Generally, pain increased immediately following separator placement and reached a peak at 24 h and then subsided until 48 h. There was no significant change in the level of pain perceived at rest over time. The results of the paired *t* test showed significant difference between T0 and T1 (*P* = 0.025), T1 and T2 (*P* = 0.005), and T2 and T3 (*P* = 0.001) while fitting posterior teeth and between T0 and T1 (*P* = 0.031), T1 and T2 (*P* = 0.003), T2 and T3 (*P* = 0.001) and T3 and T4 (*P* = 0.011) in chewing function.
2. *Ibuprofen.* Subjects experienced significant increase in the level of pain from 2 h after separator placement until 24 h at rest (T_1_–T_2_ (*P* = 0.038), T_2_–T_3_ (*P* = 0.001)). During the two other functions, patients experienced increased level of pain until its peak at 48 h. The paired *t* test showed significant increase between T_2_–T_3_ (*P* = 0.001) and T_3_–T_4_ (*P* = 0.001).
3. *Meloxicam.* The mean pain score increased immediately after separator placement and reached the peak at 24 h at rest. There was a significant increased only between T_1_ and T_2_ (*P* = 0.001); however, the difference between T_2_ and T_3_ (*P* = 0.061) tended to be significant during this function. During the fitting of posterior teeth, the mean pain score increased immediately after separator placement until the peak at 24 h (T_0_–T_1_ (*P* = 0.011), T_1_–T_2_ (*P* = 0.001), T_2_–T_3_ (*P* = 0.002)). In the chewing function, pain increased immediately after separator was placed until 48 h. Significant differences were found between T_0_–T_1_ (*P* = 0.024), T_1_–T_2_ (*P* = 0.001), and T_2_–T_3_ (*P* = 0.002)].

## Discussion

In this study, the effect of preemptive administration of acetaminophen, ibuprofen, and meloxicam in controlling post-separator pain was evaluated and compared using visual analog scale (VAS). VAS is generally accepted as a reliable and valid instrument for measuring acute and chronic pain, and is more sensitive for measuring positive responses to treatment compared to verbal descriptors [14, 36]. Our results indicated that there was no significant difference among three analgesics when administered 1 h prior to separator placement. Generally, pain increased immediately after separators were placed and in most cases reached a peak at 24 h. This result is in accordance to what has been reported in most of the previous studies [9, 11, 12, 21].However, slight increase in pain was observed after 24 h in meloxicam group in chewing function and in ibuprofen group when fitting posterior teeth. These results are comparable to those of Law et al. and Farzanegan et al. for 400 mg ibuprofen [12, 21]. Law et al. showed no alleviation in pain after 24 h “when fitting front teeth together,” and Farzanegan reported no reduction in pain levels after 24 h “when chewing” [12, 21]. It may be attributed to the blood level of medication not reaching its optimum to reduce pain efficiently in this interval.

Similar to other studies evaluating orthodontic pain level, the greatest reported pain occurred on chewing rather than at fitting posterior teeth or at rest [9, 11, 12, 21, 29]. It is not surprising because the orthodontic pain is the result of compression, inflammation, and edema in the periodontal ligament, and there is greater compression during function in the periodontal ligament (PDL) [21, 29].

Our results showed no difference between acetaminophen and ibuprofen when administered 1 h before the procedure. These findings are similar to those of Bird et al. that used single pretreatment dose of ibuprofen (400 mg) and acetaminophen (650 mg) 1 h prior to separator placement [14]. However, Patel et al. and Bradley et al. reported that ibuprofen was more effective than acetaminophen in post-separator pain control [13, 17]. It may be attributed to the administration of the follow-up doses of medication in the two latter studies. Patel et al. administered 400 mg ibuprofen or 650 mg acetaminophen 1 h before and 3 and 7 h after separator placement [17]. Bradley et al. used 400 mg ibuprofen or 1 g acetaminophen 1 h before and 6 h post-treatment [13].

Bird et al. observed a decrease in pain 2 to 3 h after separators were placed in ibuprofen (400 mg) and acetaminophen (650 mg) group when used 1 h prior to the treatment [14]. Although this trend was not found in our study, but during all the masticatory functions in the ibuprofen group and at rest in the meloxicam group, there was no significant increase in the mean pain scores until 2 h. A possible explanation could be that Bird’s study was performed on a different age range (9 to 19 years) compared to our study (≥15 years) and thus might have required lower doses of medication to reach its optimum efficacy.

Our results showed no significant difference between preoperative administration of meloxicam and the two other medications in post-separator placement pain control. To the best of our knowledge, no other studies have assessed the effectiveness of meloxicam in orthodontic pain reduction, so there is no previous report for comparison. Young et al. conducted a study on valdecoxib (a selective COX_2_ inhibitor) and showed that preemptive administration of the medication followed by five postoperative doses could effectively control post archwire placement pain [29]. It should be noted that valdecoxib has been removed from US and European markets because of the increased risk of cardiovascular events and skin reactions [29]. Bruno et al. showed that a single preoperative dose of lumiracoxib did not significantly reduced post-separator pain [30]. Nekoofar et al. proposed that there was no significant difference between meloxicam (15 mg), piroxicam (20 mg), and placebo in reducing postoperative endodontic pain when administered after treatment [27]. Aoki et al. reported that premedication with 10 mg meloxicam could reduce postsurgical pain in patients after lower third molar extraction [33]. Calvo et al. reported that pain levels after lower third molar extraction not requiring osteotomy can be successfully controlled by a single dose regimen of 7.5 mg meloxicam once daily [32].

This is clearly shown that pain during orthodontic treatment is related to inflammatory responses in the PDL [1].Orthodontic forces produce ischemia and inflammation in compressed areas that lead to the release of high levels of mediators such as prostaglandin in the PDL [1, 2]. COX_2_ is the main isoenzyme in the production of pro-inflammatory prostaglandin and also plays an important role in centrally generated hypersensitivity process [28, 33].

Meloxicam is a relatively COX_2_ inhibitor with more inhibitory effects on COX_2_ than COX_1._ Via this inhibitory effect on COX_2_, it could be effective in orthodontic pain control. In addition, as it was already mentioned, COX_1_ inhibition is responsible for the adverse gastric effects of non-selective NSAIDs [25]. It has been shown that meloxicam doses ≤15 mg decreased the incidence of gastrointestinal side effects such as perforation, ulceration, and bleeding than non-selective NSAIDs [37]. As mentioned before, there has been an increased concern regarding the risk of cardiovascular thrombotic event associated with the administration of selective NSAIDs [9, 30]. However, it seems that meloxicam is relatively safer compared to other medications of this class of NSAIDs; specifically in lower doses such as what was used in the current study (a single 7.5 mg), the risk of cardiovascular events may be very low [33, 35].

Prostaglandins play an important role in stress-related bone remodeling [38], and the potential effects of NSAIDs on the rate of orthodontic tooth movement have been considered. Acetaminophen, an NSAID from para-aminophenol family, differs from the majority of selective and non-selective NSAIDs; it does not inhibit or slightly inhibits the formation of prostaglandins and thus does not affect orthodontic tooth movement [39, 40]. It has been shown that meloxicam does not affect orthodontic tooth movement after 2 weeks of administration via drinking water in Wistar rats and seems to be safer than other selective NSAIDs [39]. There is no clear evidence on long-term effects of meloxicam on the rate of orthodontic tooth movement, and it could be an area of interest for future studies. In the absence of comprehensive study, it needs to be prescribed with caution.

In contrast to the generally accepted concept that females have greater perception of pain and lower pain threshold than males, we found no significant difference in pain experience between males and females [2, 4]. This finding is similar to several other studies that have investigated orthodontic pain [16–18, 41].

At the end, it should be noted that the pain is a subjective sensation which can be influenced significantly by factors such as cultural background, previous traumatic experience, sex, age, and psychological factors [1, 4, 7, 8, 17, 42]. We expected that the large sample volume used in the present study could offset the effects of these variables on our results.

In the present study, we compared the effect of a single preemptive dose of meloxicam with ibuprofen and acetaminophen in the control of the separator orthodontic pain. It can be suggested that a comparison between the pre- and post-administration of meloxicam and conventional analgesics be carried out in future studies, but the effect of long-term use of this drug on the rate of orthodontic tooth movement should be noted.

## Conclusions

Our findings suggest preoperative administration of meloxicam (7.5 mg) was as effective as acetaminophen (650 mg) and ibuprofen (400 mg) to control the post-separator pain. However, acetaminophen can be considered as the treatment of choice due to the fact that it does not cause gastrointestinal (GI) ulcers and does not affect the rate of tooth movement. Considering the low GI toxicity, meloxicam can be recommended as a good alternative for those patients who cannot take other NSAIDs or acetaminophen.
